# Miniature rose alleviates inflammatory bowel disease in mice by modulating gut microbiota, inhibiting TLR4, enhancing tight junction proteins, and promoting metabolism

**DOI:** 10.3389/fmicb.2025.1721294

**Published:** 2026-01-06

**Authors:** Jiaying Wu, Xuwen Mao

**Affiliations:** 1 College of Pharmacy, Urumqi, China; 2 Xinjiang Key Laboratory of Biopharmaceuticals and Medical Devices, Xinjiang Medical University, Urumqi, China

**Keywords:** inflammatory bowel disease, gut microbiota, metabolomics, miniature rose aqueous extract, intestinal barrier

## Abstract

**Introduction:**

Inflammatory bowel disease (IBD) is a chronic, relapsing inflammatory disorder of the gastrointestinal tract. Its pathogenesis is complex and not fully understood, so it remains incurable. Traditional Chinese medicine (TCM) attracts attention as a potential option. It offers multi-target actions. This study investigated the protective effects of an aqueous extract of Miniature Rose (MR), a medicinal plant from Xinjiang, in a dextran sulfate sodium (DSS)-induced murine model of IBD. We focused on gut microbiota, intestinal barrier integrity, and metabolic homeostasis.

**Methods:**

IBD was induced in mice by DSS, followed by intervention with different doses of aqueous MR extract. A multi-faceted approach incorporating 16S rRNA sequencing, non-targeted metabolomics, immunohistochemistry, and ELISA was used to evaluate the effects of MR on gut microbiota composition, fecal metabolic profiles, intestinal barrier protein expression, and the expression of the inflammatory proteins TLR4 and TLR9.

**Results:**

Treatment with aqueous MR extract markedly alleviated DSS-induced colitis. MR improved intestinal barrier integrity by upregulating the tight junction proteins Occludin (OCC) and Zonula Occludens-1 (ZO-1), while concurrently downregulating TLR4 and TLR9. MR administration also markedly modulated the gut microbiota, increasing the relative abundance of beneficial genera (*Bacteroides* and *Alloprevotella*) and decreasing the abundance of the pathobiont *Erysipelatoclostridium*. In addition, MR mitigated the metabolic dysregulation observed in DSS-induced colitis.

**Conclusion:**

MR ameliorates DSS-induced colitis through a multifaceted mechanism that involves coordinated regulation of the gut microbiota, restoration of the intestinal barrier, inhibition of inflammatory protein expression, and correction of metabolic dysregulation. These findings highlight the potential of MR as a multi-target therapeutic candidate and provide an experimental basis for its further preclinical and clinical evaluation in IBD.

## 1 Introduction

Inflammatory bowel disease (IBD) is a chronic disorder of the gastrointestinal tract, including ulcerative colitis (UC) and crohn’s disease (CD) (Flynn and Eisenstein, 2019). Typical clinical manifestations include abdominal pain, diarrhea, and hematochezia. The global prevalence of IBD is increasing, particularly in western countries and in newly industrialized regions (Windsor and Kaplan, 2019). The etiology of IBD remains incompletely understood, and current therapeutic strategies are often limited by the development of immunological tolerance and adverse side effects. Consequently, a definitive cure remains elusive.

Traditional Chinese medicine (TCM) and its active ingredients are increasingly recognized as potential therapies for IBD (Muro et al., 2025). This study focuses on Miniature Rose (MR), a characteristic medicinal plant of the Rosaceae family found in southern Xinjiang. The essential oil of MR is notably enriched in specific components; its citronellol content is 5.6-fold higher and its geraniol content is 124.9-fold higher than that of French rose. MR has been used to promote intestinal peristalsis and inhibit inflammation (Hegde et al., 2022). LC-TOF-MS/MS analysis has identified 34 active components in MR, including flavonoids (lignanoside, chrysin, quercetin dihydrate), phenolic acids (gallic acid, ellagic acid), organic acids (citric acid, linoleic acid), and triterpenes (ivy saponins, goose deoxycholic acid), as well as other polyphenols such as di-O-galloyl-hexahydroxydiphenoyl-glucosid. Previous studies have indicated that inhibition of the TLR2 and NF-κB signaling pathways contributes to the anti-inflammatory properties of lignans (Wang et al., 2018; Zuo et al., 2021). In addition, gallic acid can be metabolized into intestinal amination products that reduce the formation of harmful ammonia, thereby ameliorating colitis associated with gut dysbiosis (Peng et al., 2024).

The intestinal flora are essential for host physiological functions, playing a crucial role in maintaining immune homeostasis and producing beneficial metabolites. In patients with IBD, this ecological balance is disrupted—known as dysbiosis—and is characterized by reduced microbial diversity and an overgrowth of pathogenic microorganisms. Marked alterations in the metabolic profile of the gut microbiota also accompany IBD progression. These metabolites, including lipids, amino acids, small peptides, nucleic acids, and organic acids, exert important regulatory effects on host immunity and metabolism (Fan and Pedersen, 2021; Adolph and Zhang, 2022). Gut dysbiosis is now recognized as a key factor in the pathogenesis of IBD (Hu et al., 2022; Li et al., 2015; Lin et al., 2023). In IBD, the combination of microbial and metabolic dysregulation exacerbates intestinal barrier damage by disrupting tight junction proteins and reducing the secretion of antimicrobial peptides, thereby triggering an aberrant intestinal immune and inflammatory response (Cai et al., 2022; Gubatan et al., 2021; Liu et al., 2022). This inflammatory response further aggravates gut dysbiosis, creating a vicious cycle that perpetuates the chronic inflammatory state characteristic of IBD (Weingarden and Vaughn, 2017; Al Bander et al., 2020). Therefore, a thorough investigation of the intricate relationships among gut microbiota, host immunity, and metabolism is crucial for elucidating the pathogenesis of IBD and for developing effective therapeutic strategies.

Therefore, we hypothesized that MR alleviates IBD by modulating gut microbiota composition through the synergistic actions of its multiple active ingredients, thereby correcting metabolic imbalances and exerting anti-inflammatory effects. To elucidate the potential therapeutic mechanisms of MR in IBD, this study utilized 16S rRNA sequencing and non-targeted metabolomics to investigate MR-induced alterations in the gut microbiota and to analyze their correlations with fecal metabolites, TLRs, and tight junction proteins. This work aimed to explore the mechanisms of MR in the context of IBD development, thereby providing experimental evidence to support its potential clinical application and to aid in the identification of novel therapeutic targets.

## 2 Materials and methods

### 2.1 Drugs and reagents

MR is defined as the dried flower buds of the branchlet rose, which is the purple-red double-flowered form (Rosarugosa f.plena Byhouwer) of the rose species in the family Rosaceae (Rosarugosa Thunb.). MR crude material was purchased from Xinqikang Pharmaceutical Co., Ltd. (Urumqi, Xinjiang, China). The extract was prepared from this single rose cultivar and did not contain a mixture of different Rosa species. The dried material was ground to a powder in our laboratory and used to prepare the aqueous MR extract. For the preparation of the aqueous extract, MR powder was soaked in water for 30 min and then reflux-extracted at 80 °C. This extraction process was repeated three times. The collected extracts were pooled and concentrated to obtain a stock solution equivalent to 1 g/mL crude drug. For experimental use, this stock solution was diluted with water to the required concentrations. Gallic acid and chrysin were identified as the primary components in the aqueous MR extract.

The following primary polyclonal antibodies (pAb) were purchased: anti-ZO-1 and anti-OCC (Thermo Fisher Scientific), anti-TLR4 (Novus Biologicals), and anti-TLR9 (Bioss Antibodies). Dextran sulfate sodium (DSS) was obtained from Macklin (Shanghai, China), and dexamethasone (DEX) was obtained from Shanghai Yuanye Biotechnology Co., Ltd. (Shanghai, China).

### 2.2 Animal husbandry and ethical statement

Sixty male C57BL/6 J mice, 6–8 weeks of age and weighing 20–24 g, were obtained from the Animal Experimentation Center of Xinjiang Medical University (Xinjiang, China). The animals were acclimated for 1 week under controlled environmental conditions, including 21 ± 2 °C, 40–45% relative humidity, and a 12 h light/dark cycle.

### 2.3 IBD model induction and treatment protocol

Mice were randomly divided into six groups: Control group, DSS group, DEX (0.4 mg/kg) group, MR-L (25 mg/kg) group, MR-M (50 mg/kg) group, and MR-H (100 mg/kg) group. IBD was induced in all groups except the Control group. To induce the IBD model, mice were given free access to a 3% DSS solution for 10 consecutive days. Changes in body weight and fecal characteristics were observed and recorded daily to monitor disease progression and confirm successful model induction. The Control group and DSS group received a daily intragastric administration of 0.2 mL sterile water. The DEX group received daily intraperitoneal injections of dexamethasone (0.2 mL). The MR-L, MR-M, and MR-H groups were administered 0.2 mL/10 g of MR dry extract solution intragastrically. On day 11, the mice were anesthetized intraperitoneally with pentobarbital sodium (100–150 mg/kg) and subsequently sacrificed. The length and weight of the colon were measured. One portion of the colon tissue was fixed in polyformaldehyde, and the remaining portion was preserved at −80 °C. Fresh fecal samples were also collected and stored at −80 °C. The severity of colitis in each group was evaluated by monitoring changes in body weight, fecal characteristics, and bleeding, and by calculating the disease activity index (DAI) score (Pan et al., 2025).

### 2.4 Intestinal permeability assay

Following the final treatment, mice were deprived of food and water for 4 h. Subsequently, all mice received fluorescein isothiocyanate-dextran (FITC-DEX; 1 mg/mL) via oral gavage. Three hours after gavage, blood samples were collected from the retro-orbital sinus into heparinized tubes. Plasma was obtained by centrifuging the samples for 15 min, and the supernatant was collected. The fluorescence intensity of FITC-DEX in plasma was quantified using a spectrophotometer (excitation at 490 nm), and the corresponding concentration was used to assess intestinal permeability.

### 2.5 Histological analysis

Formalin-fixed colon tissues were processed, embedded in paraffin, and sectioned into 5-μm-thick slices. The sections were stained with hematoxylin and eosin (H&E) and examined under a light microscope to evaluate histological alterations in the colonic tissue.

### 2.6 ELISA assay

The serum levels of TNF-α, IL-6, CXCL-1, and MPO were measured using commercially available ELISA kits (Nanjing Jiancheng Bioengineering Institute, Nanjing, China), according to the manufacturer’s instructions.

### 2.7 Immunohistochemistry

Colon tissue sections were dewaxed and rehydrated through a graded ethanol series, followed by antigen retrieval and rinsing with phosphate-buffered saline (PBS). The sections were blocked with 10% goat serum and subsequently incubated with primary antibodies at 4 °C overnight and then with secondary antibodies at 37 °C for 30 min. Immunostaining was performed using 3,3′-diaminobenzidine (DAB) as the chromogen, followed by counterstaining with hematoxylin, dehydration, and mounting. The expression levels of TLR4, TLR9, OCC, and ZO-1 proteins were examined microscopically, and representative images were captured. Image processing was conducted using ImageJ software, with all groups subjected to identical standardized protocols. To quantify specific staining, the H-DAB deconvolution method was applied to separate DAB (positive signal) from hematoxylin. For each group, the DAB channel was analyzed using a consistent threshold setting. The ratio of integrated optical density (IntDen) to DAB-positive area (Area) was calculated as the primary metric of protein expression. Data were normalized relative to the control group, and eight randomly selected fields of view were analyzed per tissue section from each mouse.

### 2.8 Fecal microbial DNA extraction

Microbial DNA was isolated from fecal samples using the cetyltrimethylammonium bromide (CTAB) technique (Zhang et al., 2024). The concentration and purity of the extracted DNA were evaluated using a UV spectrophotometer. The purified DNA was then used as a template for PCR amplification of the 16S rRNA gene for subsequent high-throughput sequencing.

### 2.9 Illumina sequencing

The V3-V4 region of the 16S rRNA gene was sequenced using the Illumina MiSeq platform (PE 2 × 250 bp). Paired-end sequencing data were acquired, and adapter and barcode sequences were estimate. Following splicing and quality filtration, the DADA2 algorithm was employed using qiime dada2 denoise-paired for length filtration and denoising, producing ASV feature sequences and relative abundance tables. The ASV sequence file was annotated using the SILVA database (Release 138, https://www.arb-silva.de/documentation/release138/) and the NT-16S database, with a confidence level of 0.7 applied for taxonomy classification.

### 2.10 Bioinformatic analysis

Alpha diversity (*α*-diversity) within each sample was evaluated using multiple indices, including observed species, Shannon index, Simpson index, Chao1, Good’s coverage, and Pielou’s evenness. Microbial community composition at different taxonomic levels was visualized using percentage-stacked bar plots generated with the Wekemo Bioincloud platform (Gao et al., 2024). LEfSe was used to identify differentially abundant microbial taxa that could serve as potential biomarkers distinguishing the experimental groups. The relationships between key gut microbiota and host parameters were analyzed, and the results were displayed as correlation heatmaps using Wekemo Bioincloud and Canoco 5 software (Gao et al., 2024). Additional correlation heatmaps were generated using the online platform at www.bioinformatics.com.cn (Tang et al., 2023). Multi-group comparisons for general statistical analysis of non-microbiota data were conducted using the Kruskal–Wallis test, and Fisher’s exact test was applied to categorical data where appropriate. A *p*-value<0.05 was considered statistically significant.

### 2.11 Untargeted metabolomic analysis

Sample preparation: Metabolites were extracted from 100 mg fecal samples by adding 1 mL of methanol–water (4:1, v/v). The mixture was sonicated for 10 min and then centrifuged at 3,000 rpm for 15 min at 4 °C. The resulting supernatant was collected for UPLC-ESI-MS/MS analysis.

UPLC-ESI-MS/MS analysis: Chromatographic separation was performed on a UPLC system using an Acquity UPLC^®^ HSS T3 column (2.1 × 100 mm, 1.8 μm; Waters, Milford, MA, USA). The system operated with a column temperature of 40 °C, a flow rate of 0.3 mL/min, and an injection volume of 1 μL. The mobile phases consisted of (A) 0.1% formic acid in water and (B) 0.1% formic acid in acetonitrile. The gradient elution program was as follows: 0–2 min, 0% B; 2–6 min, linear increase from 0 to 48% B; 6–10 min, linear increase from 48 to 100% B; 10–12 min, hold at 100% B; 12–12.1 min, linear decrease from 100 to 0% B; 12.1–15 min, hold at 0% B for re-equilibration. Raw data were processed using MS-DIAL software (MSDIAL ver. 4.9.221218, Windows x64) for peak alignment, retention time correction, and peak area extraction.

Mass spectrometry was conducted using a mass spectrometer with an electrospray ionization (ESI) source operating in both positive (ESI^+^) and negative (ESI^−^) ion full-scan modes. The principal source parameters were as follows: ion spray voltage, 5,500 V; source temperature, 550 °C; curtain gas pressure, 241 kPa; collision gas, medium.

### 2.12 Statistical analysis

Statistical analyses were conducted using GraphPad Prism version 9. All continuous data are expressed as the mean ± standard error of the mean. Comparisons between two groups for normally distributed data were performed using an unpaired Student’s *t*-test. One-way analysis of variance (ANOVA) was used for comparisons among three or more groups, followed by Tukey’s *post-hoc* test. A two-way ANOVA with Bonferroni’s *post-hoc* test was used to assess the effects of two independent variables on a measured outcome. The nonparametric Kruskal–Wallis test was used to compare multiple groups when the data did not follow a normal distribution. A *p*-value below 0.05 was deemed statistically significant. Figures were produced using the same software.

## 3 Results

### 3.1 MR alleviates DSS-caused colitis in mice models

**Figure 1:**
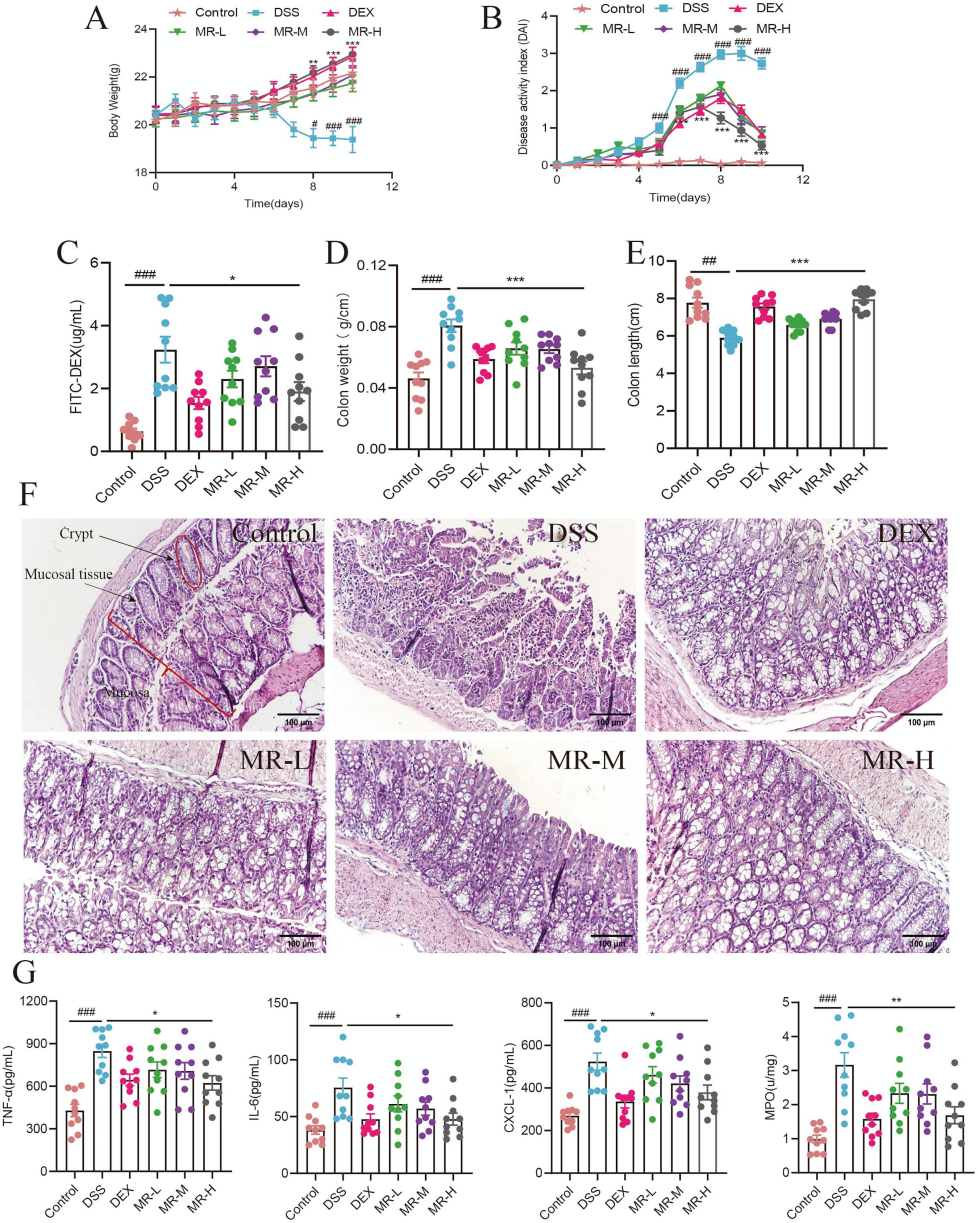
MR mitigates DSS-induced colitis in mice. **(A)** Changes in body weight over time in each group during the experimental period. **(B)** Disease activity index (DAI) scores recorded daily. **(C)** Intestinal permeability assessed by the plasma concentration of FITC-dextran (FITC-DEX). **(D)** Colon weight € Colon length at sacrifice. **(F)** Representative H&E-stained colon sections from each group (scale bar = 100 μm). **(G)** Serum concentrations of TNF-α, IL-6, CXCL-1 and MPO measured by ELISA. #*P* < 0.01, ##P < 0.05, ###*P* < 0.001 vs Control, *P < 0.01, **P < 0.05, ***P < 0.001 vs DSS. Mean ± SEM is used to express the data.

Compared with the Control group, DSS-treated mice exhibited typical features of colitis, including a significant decrease in body weight (*p* < 0.05 or *p* < 0.001), significant increases in DAI (*p* < 0.001), FITC-DEX levels (*p* < 0.001), and colon weight (*p* < 0.001), as well as a significant shortening of colon length (*p* < 0.01). Relative to the DSS group, MR treatment attenuated these changes, with the most significant effect observed in the MR-H group (*p* < 0.05 or *p* < 0.001). For all these parameters, the MR-H group showed no significant differences compared with the DEX group (Figures 1A–E). H&E staining showed that, compared with the Control group, the DSS group displayed disrupted crypt architecture, mucosal loss, and extensive inflammatory cell infiltration. In contrast, all MR-treated groups exhibited partial restoration of crypt structure, more orderly mucosal arrangement, and reduced inflammatory cell infiltration relative to the DSS group. Compared with the DEX group, mucosal tissue in the MR-L and MR-M groups showed only partial recovery, whereas the MR-H group exhibited more regularly arranged mucosa and better crypt restoration (Figure 1F). Assessment of pro-inflammatory cytokines revealed that serum levels of TNF-α (*p* < 0.001), IL-6 (*p* < 0.001), MPO (*p* < 0.001), and CXCL-1 (*p* < 0.001) were significantly higher in the DSS group than in the Control group. MR-H treatment significantly reduced the levels of these pro-inflammatory factors compared with the DSS group (*p* < 0.05 or *p* < 0.01). Relative to the DEX group, the MR-L and MR-M groups showed weaker reductions in these cytokines, whereas the anti-inflammatory effect of the MR-H group did not differ significantly from that of the DEX group (Figure 1G). Overall, these results indicate that MR effectively alleviated inflammatory symptoms and improved pathological manifestations in DSS-induced colitis, with the MR-H group showing the most pronounced therapeutic effect, comparable to that of the DEX group.

### 3.2 MR modulates toll-like receptor expression and enhances intestinal barrier integrity

**Figure 2:**
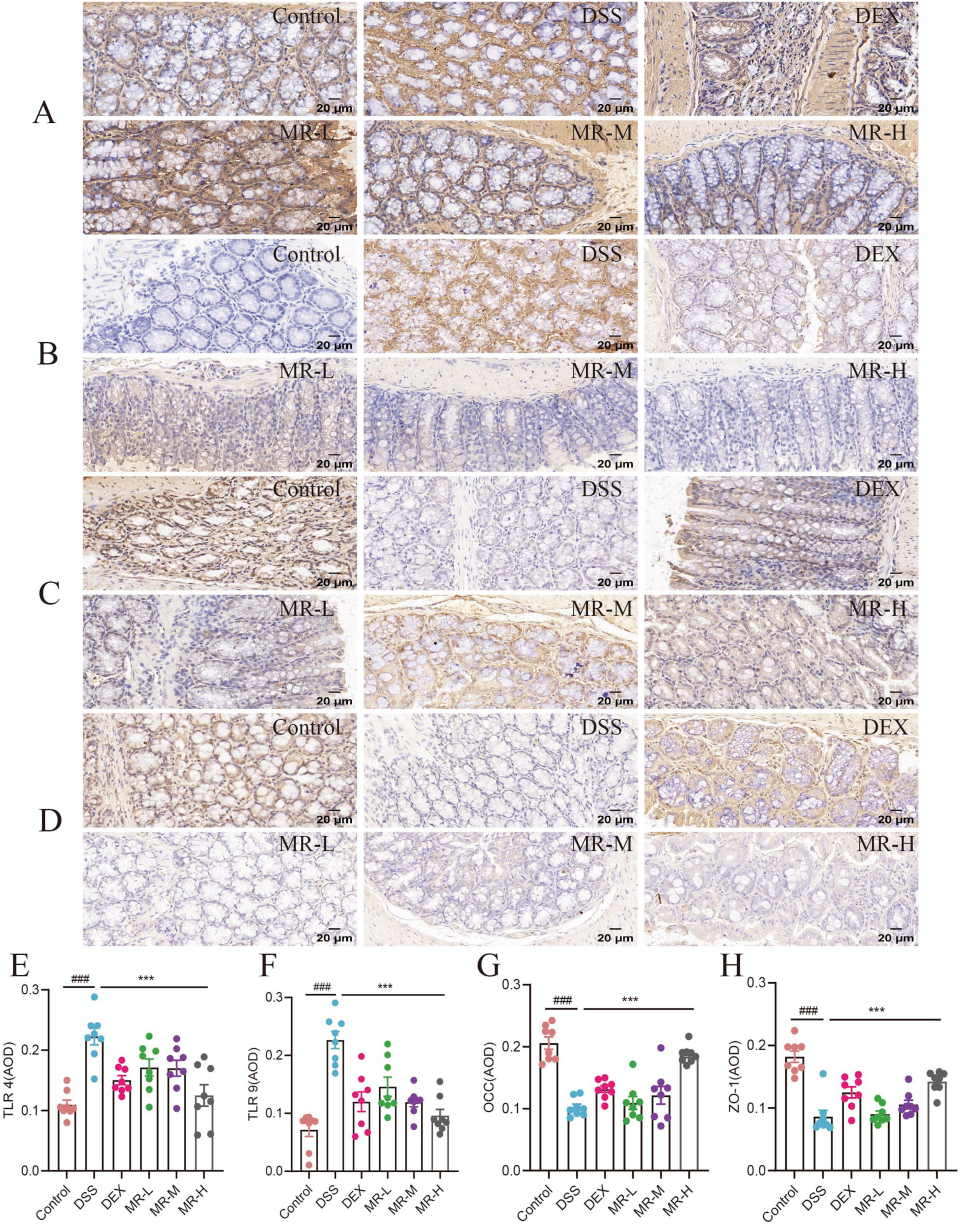
MR modulates TLR expression and enhances intestinal barrier integrity in DSS-induced colitis. A-D Representative immunohistochemical staining of colonic sections showing TLR4 **(A)**, TLR9 **(B)**, OCC **(C)**, and ZO-1 **(D)** in the Control, DSS, DEX, MR-L, MR-M, and MR-H groups (scale bar = 20 μm). **(E-H)** Quantitative analysis of average optical density (AOD) for TLR4 **(E)**, TLR9 **(F)**, OCC **(G)**, and ZO-1 (H). ### *P* < 0.001 vs Control, ***P < 0.001 vs DSS. Mean ± SEM is used to express the data.

TLR4 and TLR9 were primarily distributed in the cell membranes and cytoplasm of colonic mucosal tissues, where their expression levels correlated with the tissue’s inflammatory state. Whereas OCC and ZO-1 were mainly localized to the cell membranes, appearing as diffuse yellow granules, these proteins are critical for maintaining intercellular barrier function and cell polarity (Figures 2A–D). Quantitative analysis showed that the DSS group had significantly higher expression levels of TLR4 and TLR9 (*p* < 0.001) and significantly lower levels of OCC and ZO-1 (*p* < 0.001) than the Control group (Figures 2E–H). Following MR treatment, the MR-H group markedly reversed these changes, with significantly reduced TLR4 and TLR9 (*p* < 0.001) expression and significantly increased OCC and ZO-1 (*p* < 0.001) expression compared with the DSS group. Compared with the DEX group, the MR-L and MR-M groups showed weaker inhibition of TLR4 and TLR9 and less recovery of OCC and ZO-1, whereas the MR-H group exhibited lower TLR4 and TLR9 expression and more fully restored OCC and ZO-1 expression than the DEX group. These results indicate that MR inhibited the expression of the pro-inflammatory pathway proteins TLR4 and TLR9, increased the expression of tight junction proteins, and thereby helped maintain intestinal mucosal barrier function in IBD mice.

### 3.3 MR modulates gut microbiota dysbiosis and restores microbial diversity

**Figure 3:**
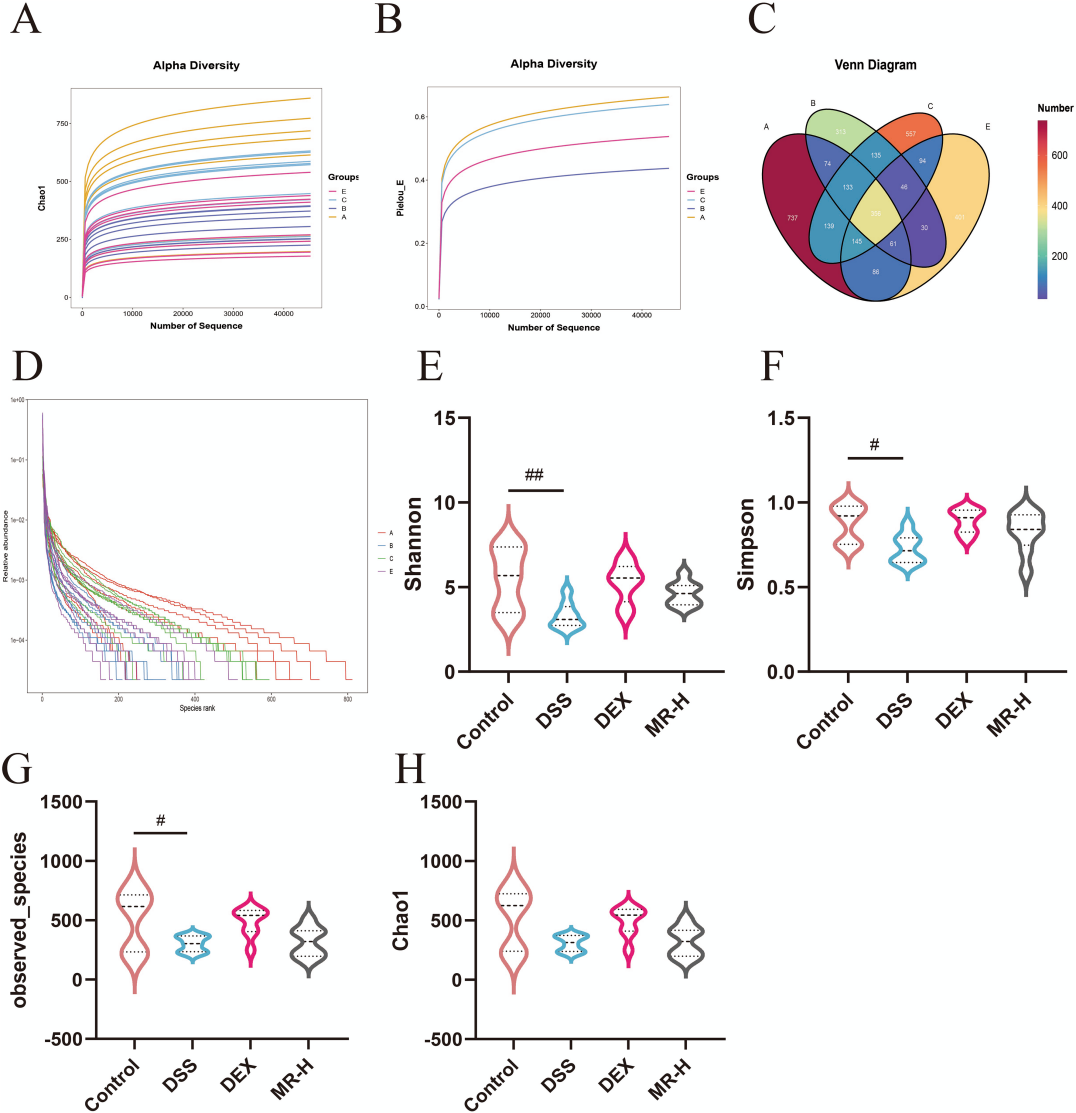
MR modulates intestinal microbiota diversity and richness in DSS-induced colitis. **(A)** Chao1 rarefaction curves showing sequencing depth and species richness for each group. **(B)** Pielou’ s evenness rarefaction curves. **(C)** Venn diagram illustrating the numbers of shared and unique ASVs among the four groups. **(D)** Rank-abundance curves depicting the relative abundance distribution of microbial taxa. **(E)** Shannon diversity index violin plots. **(F)** Simpson diversity index violin plots. **(G)** Observed species richness violin plots. **(H)** Chao1 diversity index violin plots. #*P* < 0.01, ##*P* < 0.05 vs Control. Data are presented as mean ± SEM. In panels A-D, groups A, B, C and E correspond to the Control, DSS, DEX and MR-H groups, respectively.

The rarefaction (dilution) curves for all groups tended to level off, indicating that the sequencing depth was sufficient to capture the vast majority of species present in the samples (Figures 3A,B). Venn diagram analysis showed that the total number of ASVs in the DSS group decreased sharply to 313 compared with the Control group, whereas both MR-H and DEX treatment increased ASV richness to 401 and 557, respectively (Figure 3C). Rank–abundance analysis further confirmed that both MR-H and DEX enhanced species richness, with comparable effects (Figure 3D). *α*-Diversity analysis revealed that, relative to the Control group, the DSS group showed significantly reduced Shannon (*p* < 0.01), Simpson (*p* < 0.05), and observed-species (*p* < 0.05) indices, with a decreasing trend in the Chao1 index. Compared with the DSS group, these diversity indices increased after MR-H treatment and reached levels similar to those observed in the DEX group (Figures 3E–H). These results indicate that MR treatment can help restore the overall diversity and richness of the intestinal microbiota in IBD mice.

### 3.4 MR rebalances the gut microbiota composition at the phylum level

**Figure 4:**
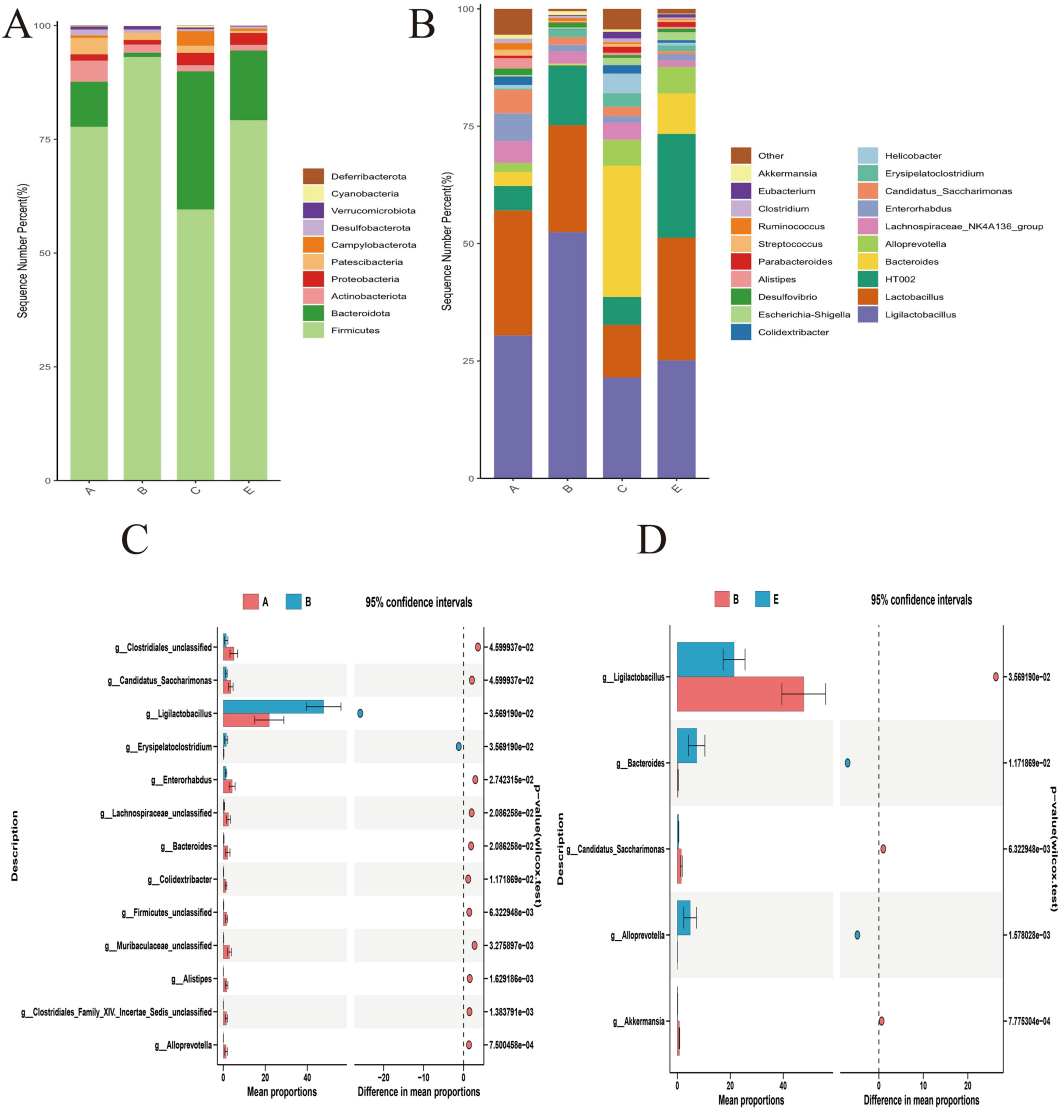
Analysis of gut microbiota composition and variations in DSS-induced colitis. **(A)** Stacked bar plots showing the relative abundance of major bacterial phyla in the Control, DSS, DEX and MR-H groups. **(B)** Stacked bar plots showing the relative abundance of major bacterial genera in the four groups. **(C)** Comparative analysis of differential genera between the Control and DSS groups. **(D)** Comparative analysis of differential genera between the DSS and MR-H groups. Only genera with statistically significant differences in relative abundance are shown (*P* < 0.05); bars indicate mean proportions and horizontal lines represent 95% confidence intervals. In panels A-D, labels A, B, C and E correspond to the Control, DSS, DEX and MR-H groups, respectively.

A total of 24 phyla, 62 classes, 128 orders, 222 families, 509 genera, and 723 species were identified across all samples. *Firmicutes* and *Bacteroidota* were the predominant phyla in all experimental groups. Marked dysbiosis was observed in the DSS group compared with the Control group, with the relative abundance of *Firmicutes* increasing from 77.72 to 93.06% and that of *Bacteroidota* decreasing from 9.91 to 0.96%. MR-H and DEX treatment partially counteracted this DSS-induced shift, with *Firmicutes* and *Bacteroidota* restored to 79.18 and 15.33%, respectively, values close to those of the Control group (Figure 4A).

At the genus level, *Ligilactobacillus*, *Lactobacillus*, *Bacteroides*, *Alloprevotella*, and *Erysipelatoclostridium* were the predominant genera across the Control, DSS, DEX, and MR-H groups. Clear alterations in gut microbiota composition were observed when comparing the DSS and Control groups (Figure 4B). Genus-level differential analysis showed that, compared with the Control group, the relative abundances of 11 genera (*p* < 0.05), including *Bacteroides* and *Alloprevotella*, were significantly decreased in the DSS group, whereas the relative abundance of *Erysipelatoclostridium* (*p* < 0.05) was significantly increased, specifically, the relative abundance of *Erysipelatoclostridium* in the DSS group increased by 0.15 times, while the relative abundances of *Lactobacillus*, *Bacteroides* and *Alloprevotella* decreased by 0.95, 7.48 and 69 times, respectively. Compared with the DSS group, the abundance of *Bacteroides* in the MR-H group recovered to 7.32%, and the abundance of *Alloprevotella* recovered to 4.84%. At the same time, the relative abundance of *Erysipelatoclostridium* in the MR-H group decreased to 1.07%, which was lower than that in the DSS group (Figures 4C,D). These results indicate that MR treatment promoted the growth of potentially beneficial bacteria in the intestines of IBD mice, suppressed the proliferation of pathogenic bacteria, and modulated the overall composition of the intestinal microbiota.

**Figure 5:**
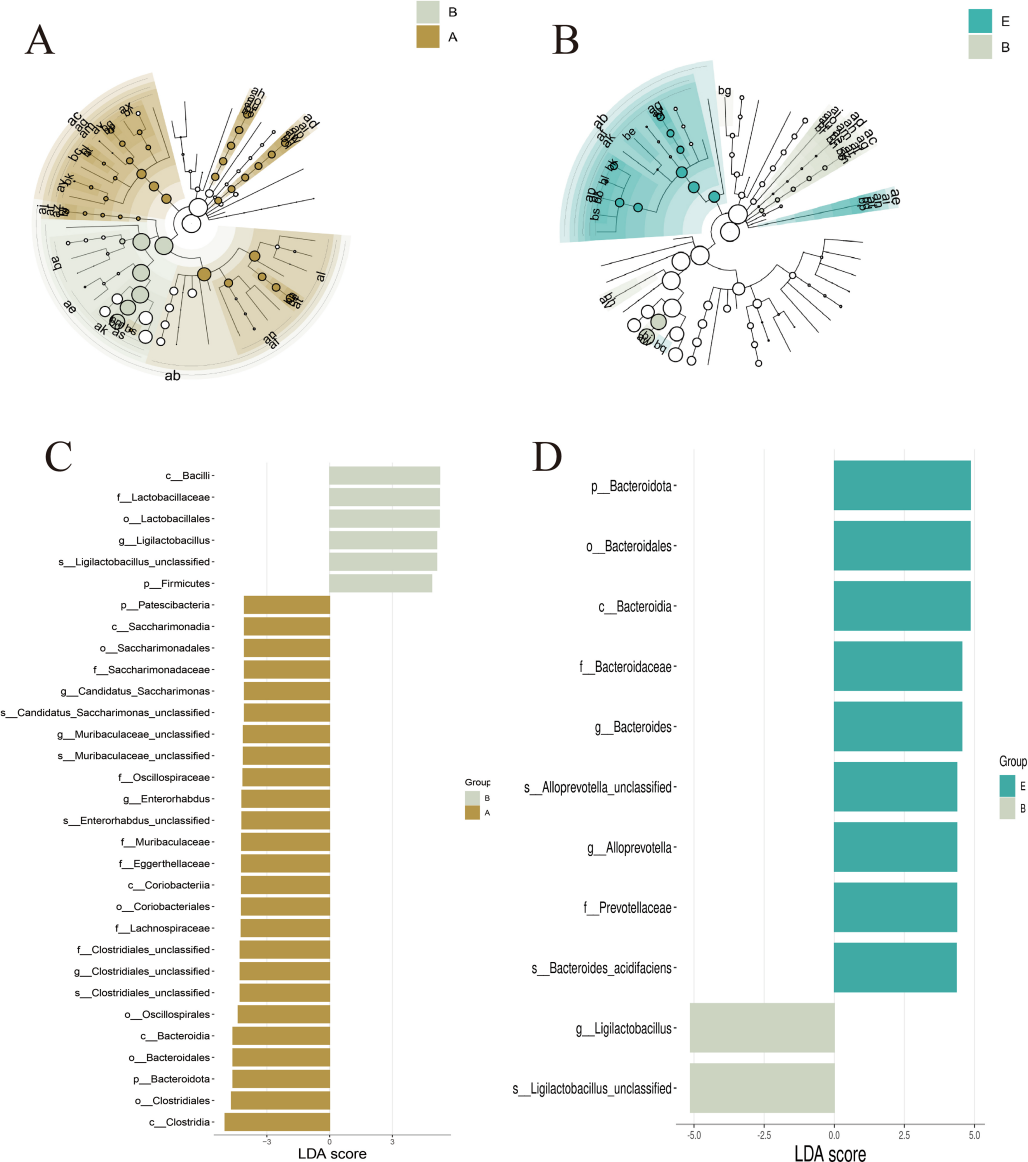
LEfSe analysis reveals distinct gut microbiota signatures among groups. **(A-B)** Cladograms generated by LEfSe showing bacterial taxa with differential 749 abundance between groups. Concentric circles represent the seven taxonomic levels from domain (center) to species (outermost). Each node corresponds to one taxon, with node size proportional to its relative abundance. Colorless nodes indicate taxa without significant differences between groups, whereas colored nodes indicate taxa that are significantly enriched in the corresponding group (A: Control vs DSS; B: DSS vs MR-H). **(C-D)** LDA score plots of taxa with significantly different relative abundances between groups. Bar colors indicate the group in which each taxon is enriched, and bar length represents the magnitude of the LDA score (C: Control vs DSS; D: DSS vs MR-H). LEfSe analysis was performed with a significance threshold of *P* < 0.05 and LDA score>3.0; only taxa meeting these criteria are shown. In panels A and B, labels A, B and E correspond to the Control, DSS and MR-H groups, respectively.

To identify key bacterial taxa that differentiated the experimental groups, LEfSe analysis was performed (LDA score>3.0, *p* < 0.05). *Ligilactobacillus* was enriched in the DSS group, whereas *Bacteroides* and *Alloprevotella* were more abundant in the MR-H group (Figures 5A–D).

### 3.5 Correlation analysis links key microbial taxa with host pathophysiological indicators

**Figure 6:**
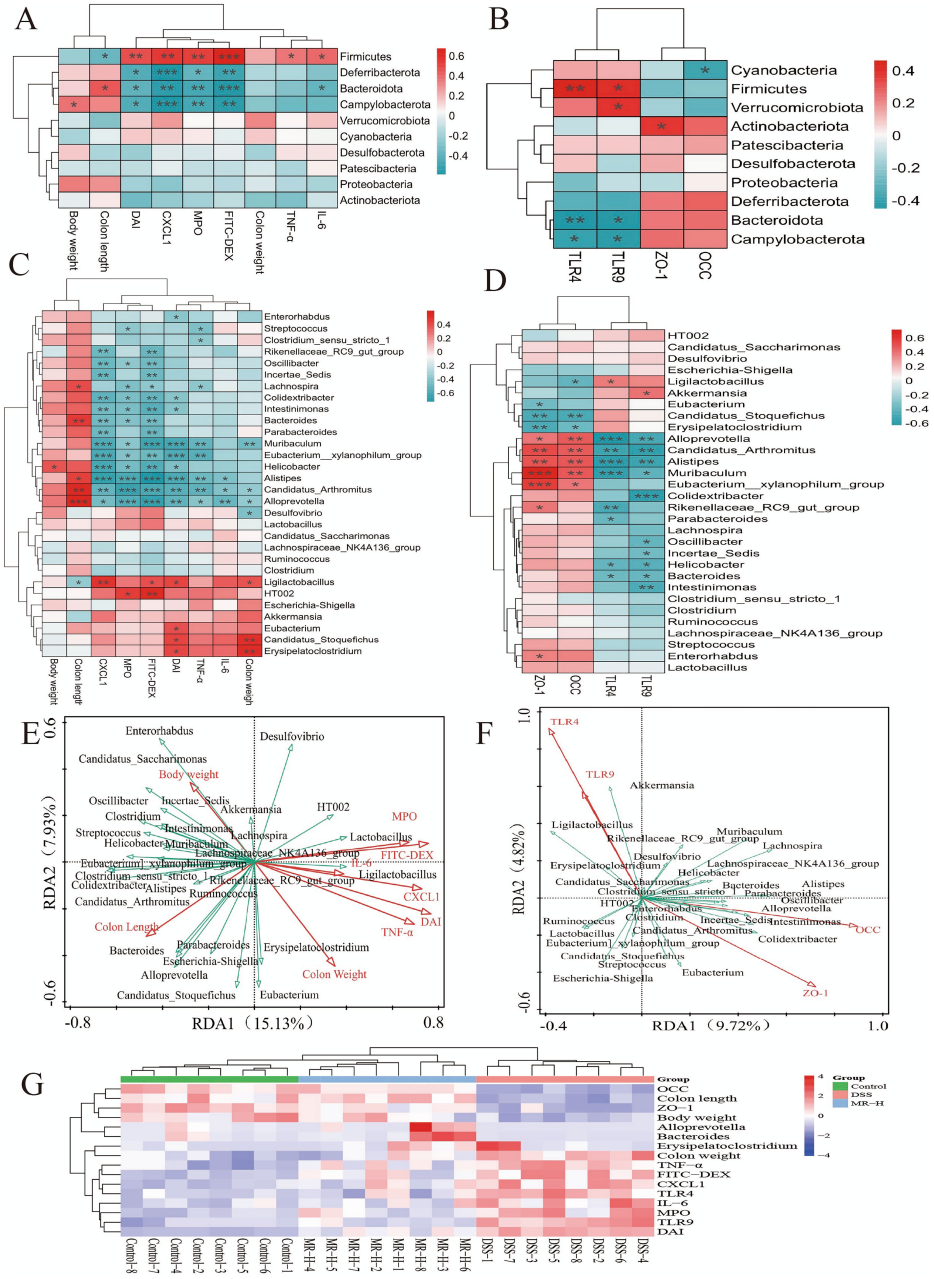
Correlation and redundancy analysis (RDA) linking gut microbiota with host pathophysiological indicators. **(A)** Correlation heatmap of phylum-level taxa with indicators of disease severity and inflammation (body weight, colon length, colon weight, DAI, FITC-DEX, TNF-α, IL-6, CXCL-1, MPO). (B) Correlation heatmap of phylum-level taxa with TLR4, TLR9, OCC and ZO-1. (C) Correlation heatmap of genus-level taxa with disease severity and inflammatory indicators. (D) Correlation heatmap of genus-level taxa with TLR4, TLR9, OCC and ZO-1. (E) RDA biplot illustrating the relationships between genus-level gut microbiota and disease/inflammatory indicators. (F) RDA biplot showing the relationships between genus-level gut microbiota and TLR4, TLR9, OCC and ZO-1. (G) Clustered heatmap summarizing correlations between representative genera and key clinical, inflammatory and barrier-related parameters across the Control, DSS, DEX and MR-H groups. In all heatmaps, red indicates positive correlations and blue indicates negative correlations, asterisks denote statistically significant correlations. *P < 0.05, **P < 0.01, ***P < 0.001.

To investigate the relationships between microbiota shifts and host pathophysiology, correlation analysis was performed. At the phylum level, the relative abundance of *Firmicutes* showed a significant positive correlation with indicators of disease severity (DAI, FITC-DEX, pro-inflammatory factors, TLR4, TLR9; *p* < 0.001 or *p* < 0.01 or *p* < 0.05) and a negative correlation with markers of intestinal health (body weight, colon length *p* < 0.05, OCC, ZO-1). In contrast, the abundance of *Bacteroidota* exhibited the opposite pattern, significantly positively correlating with health-related markers (*p* < 0.001 or *p* < 0.01 or *p* < 0.05) and negatively with disease-related markers (*p* < 0.05; Figures 6A,B). A similar trend was observed at the genus level: the abundance of *Erysipelatoclostridium* showed a significant positive correlation with disease indicators (*p* < 0.01 or *p* < 0.05), whereas *Bacteroides* and *Alloprevotella* showed a significant positive correlation with markers of intestinal health and barrier integrity (*p* < 0.001 or *p* < 0.01 or *p* < 0.05) and negatively correlated with indicators of inflammation and pathology (*p* < 0.001 or *p* < 0.01 or *p* < 0.05; Figures 6C,D).

Redundancy analysis (RDA) was performed to assess the associations between the gut microbiota and the measured indicators. In the RDA ordination plot, arrows of different colors represent distinct explanatory variables, and the angles between arrows indicate the degree of correlation: acute angles reflect positive correlations, obtuse angles reflect negative correlations, and right angles indicate no correlation. The RDA plot shows that genera such as *Bacteroides*, *Alloprevotella*, and *Akkermansia* are positively associated with intestinal health-related indicators, whereas genera such as *Erysipelatoclostridium* and *Desulfovibrio* are positively associated with inflammatory and pathological indicators (Figures 6E,F). Correlation analysis also revealed that in the DSS group, indicators of disease severity and inflammation were positively correlated with the abundance of *Erysipelatoclostridium*. Conversely, these pathological indicators were negatively correlated with indicators of intestinal health and with the beneficial genera *Bacteroides* and *Alloprevotella*. MR-H treatment altered these adverse correlation patterns (Figure 6G). These results suggest that MR-H may alleviate IBD symptoms by modulating the intestinal microbiota, promoting intestinal barrier repair, and inhibiting TLR4/TLR9-mediated inflammation.

### 3.6 MR reverses DSS-induced fecal metabolomic dysregulation

**Figure 7:**
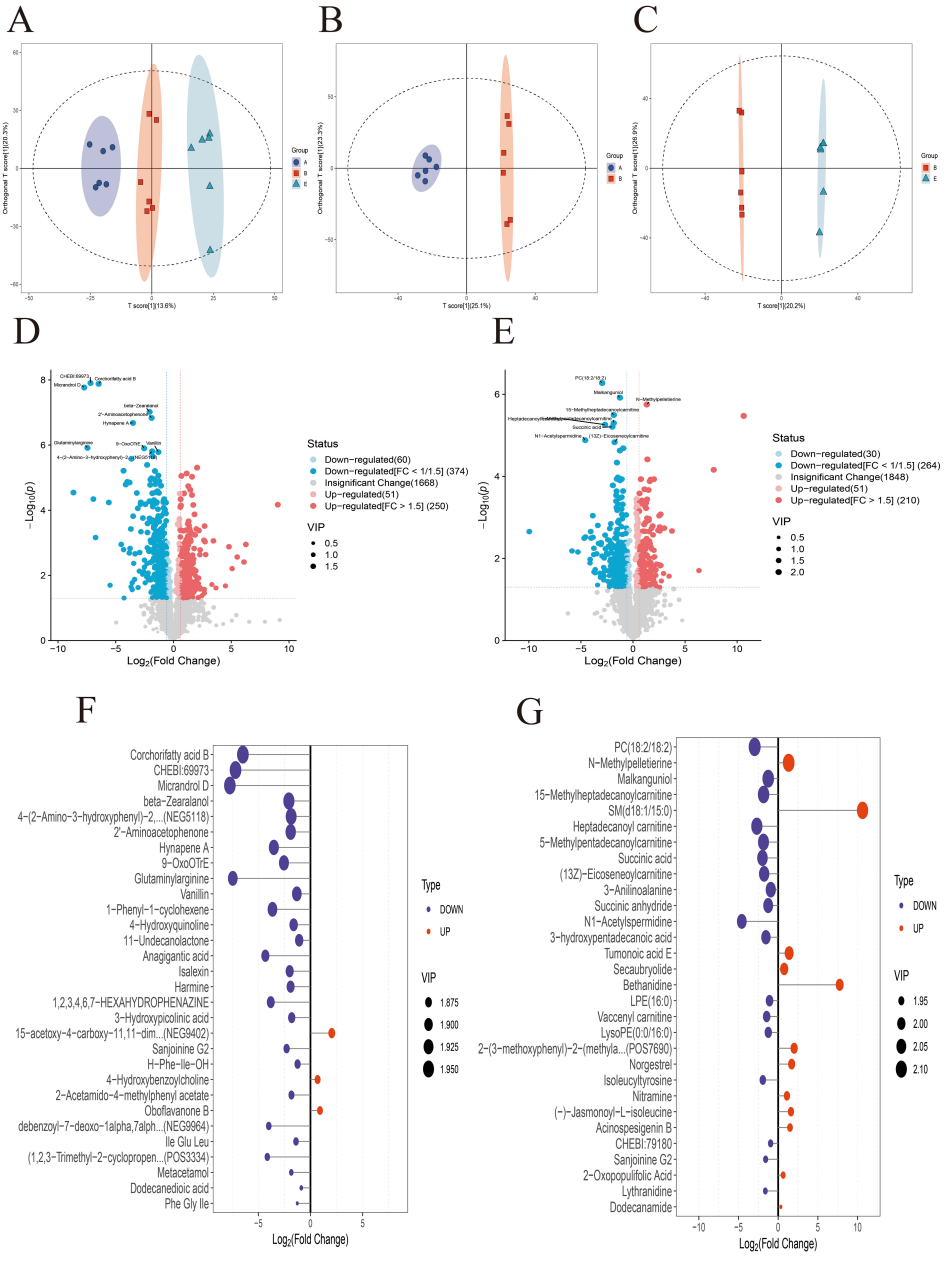
Untargeted fecal metabolomics analysis in DSS-induced colitis. **(A)** OPLS-DA score plot showing separation among the Control, DSS and MR-H groups. **(B)** OPLS-DA score plot comparing the Control and DSS groups. **(C)** OPLS-DA score plot comparing the DSS and MR-H groups. **(D)** Volcano plot of differential metabolites between the Control and DSS groups, highlighting significantly upregulated and downregulated metabolites. **(E)** Volcano plot of differential metabolites between the DSS and MR-H groups. **(F)** Variable importance in projection (VIP) plot showing key differential metabolites between the Control and DSS groups. **(G)** VIP plot showing key differential metabolites between the DSS and MR-H groups. Differential metabolites were defined as those with VIP>1.0 in the OPLS-DA model and *P* < 0.05 in univariate analysis; in the volcano plots (D-E), significantly upregulated and downregulated metabolites (FC≥1.5, *P* < 0.05) are highlighted. In the OPLS-DA score plots A-C, group labels A, B and E correspond to the Control, DSS and MR-H groups, respectively.

Multivariate analysis using orthogonal partial least-squares discriminant analysis (OPLS-DA) showed a clear separation of fecal metabolic profiles among the Control, DSS, and MR-H groups. The DSS group displayed a metabolic profile distinct from that of the Control group, whereas the MR-H group clustered closer to the Control group (Figures 7A–C). Univariate analysis, visualized by volcano plots, further illustrated these shifts. Compared with the Control group, DSS treatment was associated with widespread metabolic disturbances, with 374 metabolites (*p* < 0.05) significantly downregulated and 250 (*p* < 0.05) significantly upregulated. Relative to the DSS group, MR-H treatment was associated with significant changes in 528 metabolites, including 264 (*p* < 0.05) that were downregulated and 264 (*p* < 0.05) that were upregulated (Figures 7D,E). Analysis of differential metabolites revealed significant changes in the DSS group compared with the Control group, specifically, 27 metabolites (*p* < 0.05), including Corchorifatty acid B, 3-(phenylamino) alanine, and Micrandrol D, were significantly downregulated, whereas 3 metabolites (*p* < 0.05), such as 4-Hydroxybenzoylcholine and Oboflavanone B, were significantly upregulated. In contrast, MR-H treatment induced a distinct metabolic profile compared with the DSS group, with 18 metabolites (*p* < 0.05), including Phosphatidylcholine, Malkanguniol, and 15-Methylheptadecanoylcarnitine, significantly downregulated and 12 metabolites (*p* < 0.05), including N-Methylpelletierine and sphingomyelin (d 18:1/15:0), significantly upregulated (Figures 7F,G).

**Figure 8:**
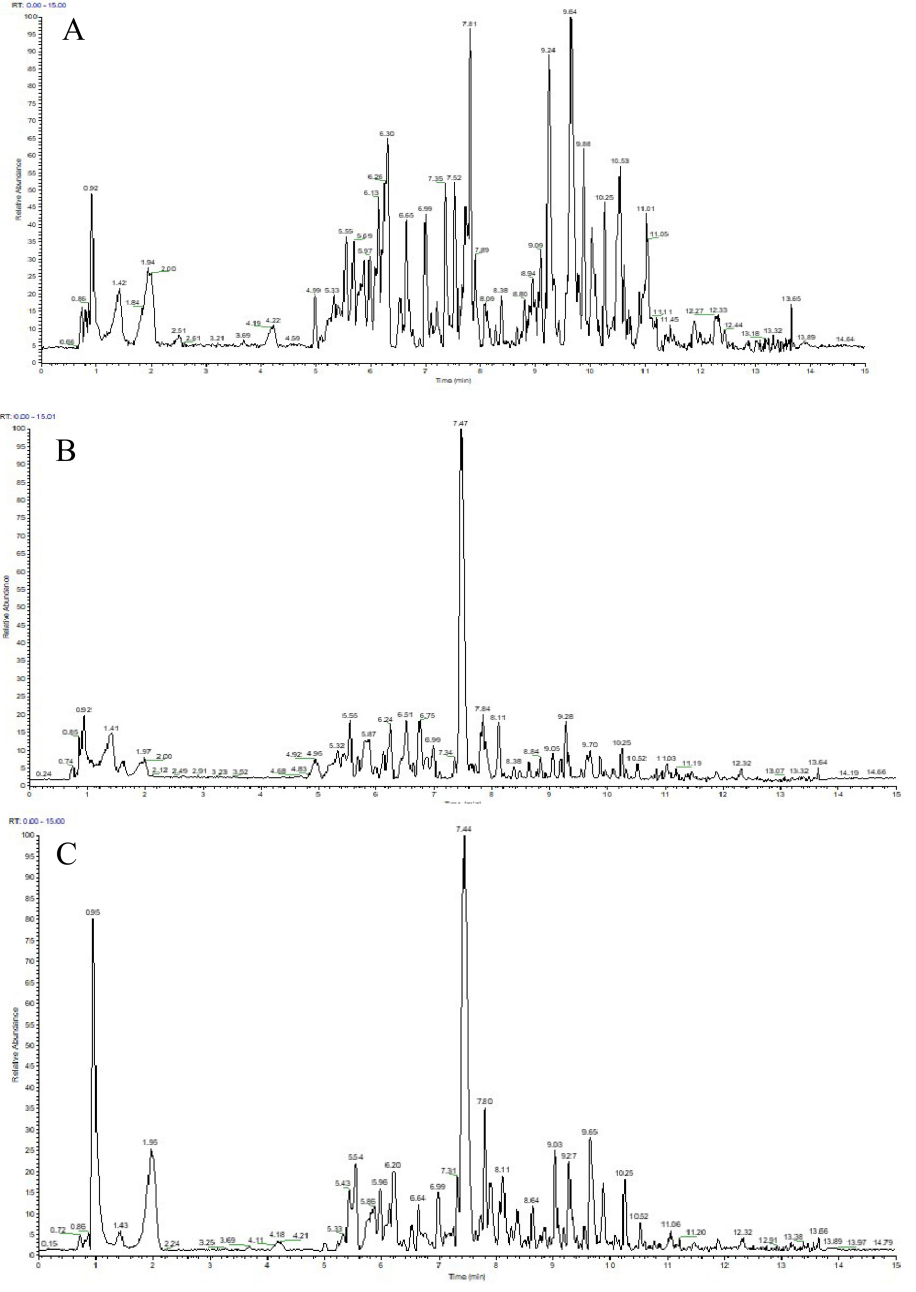
The UPLC-ESI-MS/MS figure. **(A)** Control group. **(B)** DSS group **(C)** MR-H group. Representative UPLC-ESI-MS/MS total ion chromatograms of fecal metabolites. A Control group. B DSS group. C MR-H group.

To further characterize these changes, a UPLC-ESI-MS/MS-based metabolomics analysis was performed, and representative chromatograms for the Control, DSS, and MR-H groups are shown in Figure 8. Comparative analysis of the top 10 metabolites demonstrated pronounced alterations in the DSS group relative to the Control group (Table 1), and MR-H treatment was associated with additional shifts in the metabolic profile compared with the DSS group (Table 2). Intersection analysis identified six key differential metabolites common to all three groups: Sanjoinine G2, Bethanidine, Heptadecanoyl carnitine, 2-Oxopopulifolic acid, 15-Methylheptadecanoylcarnitine, and 5-Methylheptadecanoylcarnitine (Table 3).

**Table 1:** Differentially expressed metabolites in the control and model group.

Metabolites	*m/z*	Trend	VIP	*p*
Hynapene A	323.19	DOWN	1.928	0.00000021
beta-Zearalanol	321.17	DOWN	1.954	0.00000010
Corchorifatty acid B	307.19	DOWN	1.967	0.00000001
Glutaminylarginine	301.16	DOWN	1.921	0.00000122
9-OxoOTrE	291.20	DOWN	1.927	0.00000126
15,16-bisnor-13-oxo-8 (17),11Elabdadien-19-oic acid	289.18	DOWN	1.966	0.00000001
Micrandrol D	287.17	DOWN	1.964	0.00000002
4-(2-Amino-3-hydroxyphenyl)-2,4-dioxobutanoate	222.04	DOWN	1.947	0.00000155
Vanillin	153.05	DOWN	1.921	0.00000861
2′-Aminoacetophenone	136.08	DOWN	1.935	0.00000015

**Table 2:** Differentially expressed metabolites in the model and MR-H group.

Metabolites	*m/z*	Trend	VIP	*p*
Phosphatidylcholine	782.57	DOWN	2.139	0.000001
Sphingomyelin	689.56	UP	2.110	0.000003
(13Z)-Eicoseneoylcarnitine	454.39	DOWN	1.893	0.000015
15-Methylheptadecanoylcarnitine	428.37	DOWN	2.114	0.000003
Heptadecanoyl carnitine	414.36	DOWN	0.000	2.104919
5-Methylpentadecanoylcarnitine	400.34	DOWN	2.096	0.000005
Malkanguniol	285.17	DOWN	2.122	0.000001
3-Anilinoalanine	181.10	DOWN	2.065	0.000021
N-Methylpelletierine	156.14	UP	2.129	0.000002
Succinic acid	117.02	DOWN	2.076	0.000006

**Table 3:** Differentially expressed metabolites in the control and model and MR.

Metabolites	*m/z*	Trend		VIP	*p*
		AvsB	BvsE		
Sanjoinine G2	117.02	DOWN	DOWN	1.15	0.0000003
Bethanidine	178.13	UP	UP	0.12	0.0000007
Heptadecanoyl carnitine	414.36	DOWN	DOWN	1.63	0.0000015
2-Oxopopulifolic acid	343.22	UP	UP	0.96	0.000002
15-Methylheptadecanoylcarnitine	428.37	DOWN	DOWN	1.45	0.0000035
5-Methylpentadecanoylcarnitine	400.34	DOWN	DOWN	1.51	0.0000051

**Figure 9:**
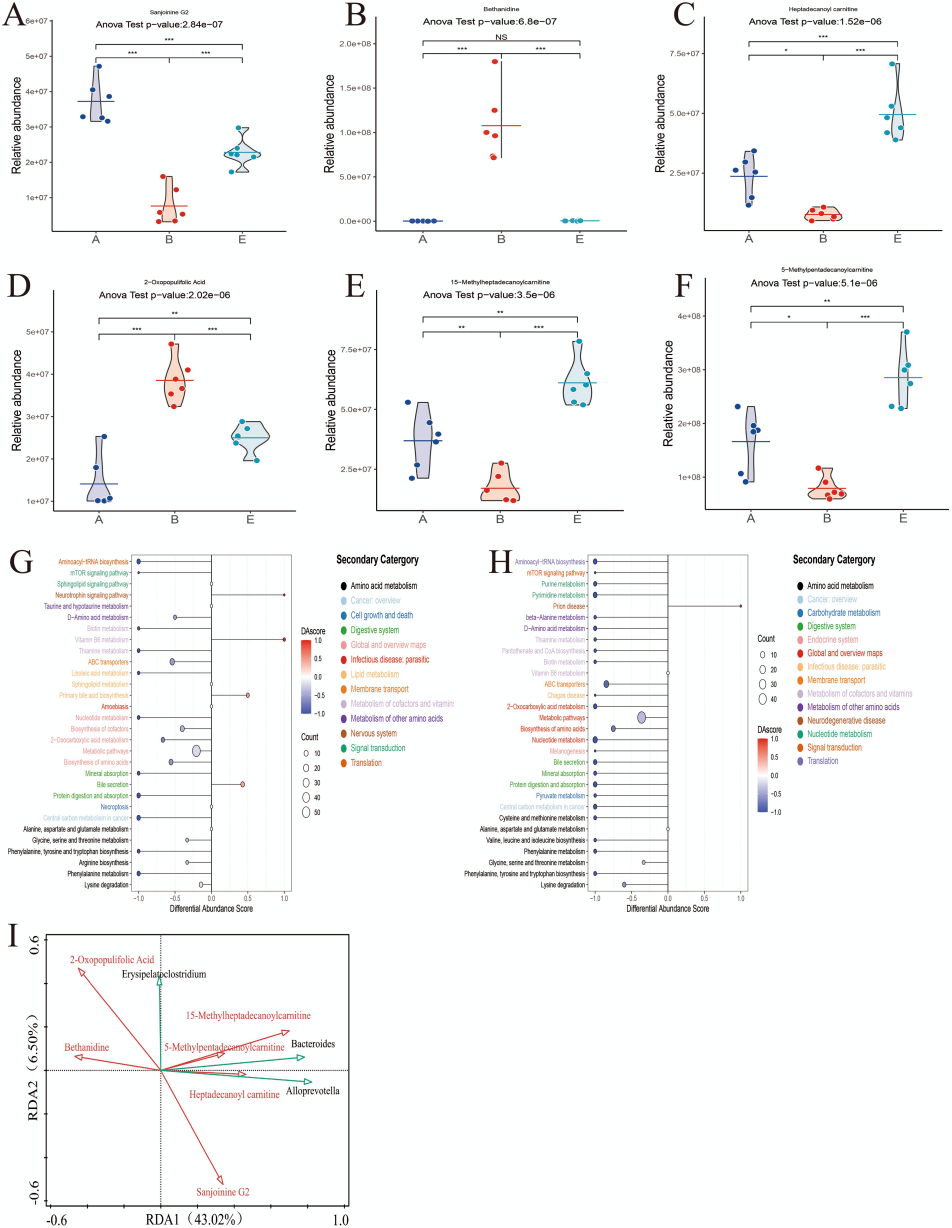
Key differential metabolites and their pathway and microbiota associations. A-F Violin plots showing the relative abundances of six shared differential metabolites 793 among the Control, DSS and MR-H groups: **(A)** Sanjoinine G2 **(B)** Bethanidine **(C)** Heptadecanoyl carnitine **(D)** 2-Oxopopulifolic acid **(E)** 15-Methylheptadecanoylcarnitine **(F)** 5-Methylpentadecanoylcarnitine. **(G)** KEGG pathway enrichment analysis of differential metabolites between the Control and DSS groups. **(H)** KEGG pathway enrichment analysis of differential metabolites between the DSS and MR-H groups. **(I)** RDA biplot illustrating the relationships between the six key metabolites and the genera Erysipelatoclostridium, Alloprevotella and Bacteroides. *P <0.05, **P < 0.01, ***P < 0.001. Data are presented as mean ± SEM. For the pathway analyses in panels G and H, differential metabolites were defined as those with a VIP>1.0 in the OPLS-DA model and a *P* < 0.05 in univariate analysis. Bubble size indicates the number of differential metabolites mapped to each pathway, and bubble color reflects the differential abundance score. Inpanels A-F, group labels A, B and E correspond to the Control, DSS and MR-H groups, respectively.

Comapred with DSS group, analysis of specific differential metabolites showed that MR-H treatment significantly increased the abundances of Sanjoinine G2, Heptadecanoyl carnitine, 15-Methylheptadecanoylcarnitine, and 5-Methylpentadecanoylcarnitine (all *p* < 0.001), while significantly decreasing the levels of Bethanidine and 2-Oxopopulifolic acid (all *p* < 0.001; Figures 9A–F). KEGG pathway analysis indicated that, relative to the Control group, the DSS group exhibited significantly dysregulation of multiple pathways: pathways including the Neurotrophin signaling pathway (*p* < 0.05) and Vitamin B6 metabolism (*p* < 0.05) were significantly enriched, whereas pathways such as Aminoacyl-tRNA biosynthesis (*p* < 0.05) and protein digestion (*p* < 0.05) and absorption (*p* < 0.05) were depleted. Compared with the DSS group, MR-H treatment significantly altered these pathway changes, with pathways such as Pyrimidine metabolism (*p* < 0.05) and Thiamine metabolism (*p* < 0.05) being downregulated and pathways including the prion disease (*p* < 0.05) pathway being upregulated (Figures 9G,H).

### 3.7 Key microbial genera strongly correlate with differentiating metabolites

RDA analysis at the genus level revealed extensive and significant associations between the gut microbiota and differential metabolites. In particular, the genera *Bacteroides*, *Alloprevotella*, and *Erysipelatoclostridium* showed strong correlations with key differential metabolites, including Sanjoinine G2, Bethanidine, 2-Oxopopulifolic acid, and several acylcarnitines (Heptadecanoyl carnitine, 15-Methylheptadecanoylcarnitine, and 5-Methylpentadecanoylcarnitine; Figure 9I and Table 4).

**Table 4:** Correlations between gut microbiota and serum metabolites.

Phylum	Genus	Metabolites	*R*	*p*
*Bacteroidota*	*Bacteroides*	Sanjoinine G2	0.1967	0.4342
		Bethanidine	−0.3328	0.1772
		Heptadecanoyl carnitine	0.3694	0.1314
		2-Oxopopulifolic acid	−0.2977	0.2302
		15-Methylheptadecanoylcarnitine	0.5563	0.0165
		5-Methylpentadecanoylcarnitine	0.2792	0.2618
*Bacteroidota*	*Alloprevotella*	Sanjoinine G2	0.3429	0.1636
		Bethanidine	−0.4178	0.0845
		Heptadecanoyl carnitine	0.3766	0.1234
		2-Oxopopulifolic Acid	−0.4198	0.0828
		15-Methylheptadecanoylcarnitine	0.5695	0.0136
		5-Methylpentadecanoylcarnitine	0.2805	0.2595
*Firmicutes*	*Erysipelatoclostridium*	Sanjoinine G2	−0.2206	0.3790
		Bethanidine	0.02413	0.9243
		Heptadecanoyl carnitine	−0.01346	0.9577
		2-Oxopopulifolic Acid	0.2014	0.4230
		15-Methylheptadecanoylcarnitine	0.07404	0.7703
		5-Methylpentadecanoylcarnitine	0.03092	0.9031

## 4 Discussion

### 4.1 MR ameliorates colitis by modulating gut microbiota and inhibiting TLR-mediated inflammation

**Figure 10:**
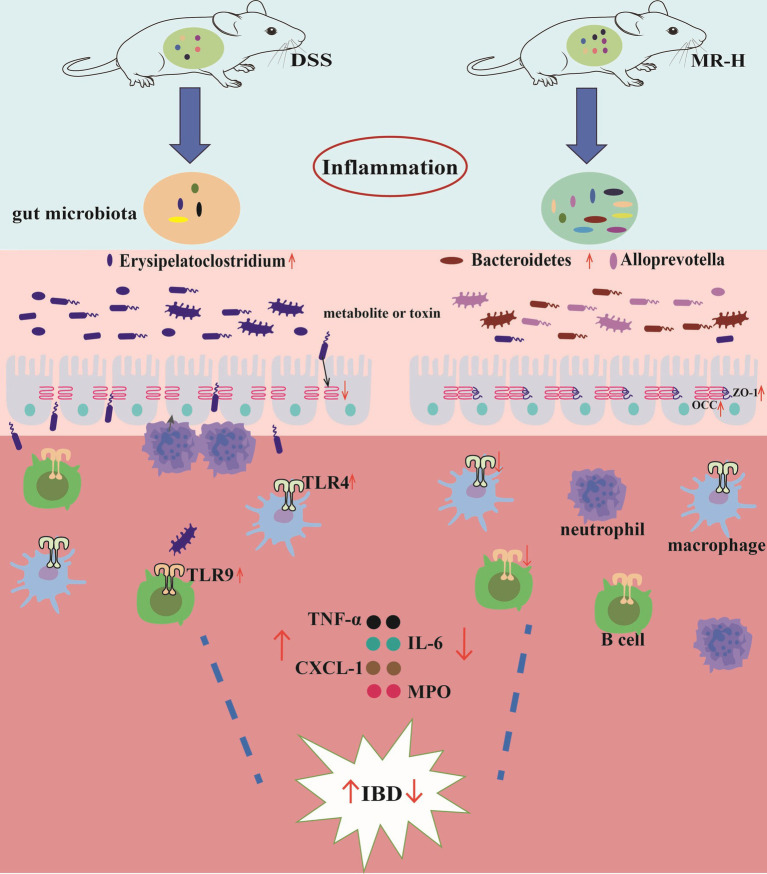
Schematic illustration of DSS-induced IBD pathogenesis and the possible partial mechanisms of MR in treating IBD. DSS-induced colitis is characterized by gut microbiota dysbiosis, disruption of the intestinal epithelial barrier (The expression levels of OCC and ZO-1 are downregulated, increased permeability), activation of TLR4/TLR9 signaling and overproduction of pro-inflammatory cytokines, leading to mucosal injury and IBD-like symptoms. MR treatment modulates gut microbiota and fecal metabolites, inhibits TLR4/TLR9-mediated inflammatory responses, restores tight junction proteins and barrier integrity, and thereby attenuates DSS-induced colitis.

Our study demonstrates that MR, a traditional Chinese medicine, effectively ameliorates DSS-induced colitis through a multifaceted mechanism involving modulation of the gut microbiota, restoration of the intestinal barrier, suppression of inflammatory responses, and correction of metabolic dysregulation (Figure 10). The pivotal role of the gut microbiota in IBD pathogenesis is well established; dysbiosis can compromise intestinal barrier integrity and trigger aberrant immune activation (Chen et al., 2023). Targeting this dysbiosis is therefore considered a key therapeutic strategy. In this context, we found that MR treatment markedly remodeled the gut microbiota by selectively suppressing potentially pathogenic bacteria while enriching beneficial microbial populations. A key observation was the expansion of *Erysipelatoclostridium* in the DSS group, which is consistent with reports in clinical IBD patients (Qiu et al., 2017; Nagayama et al., 2020; Sun et al., 2019; Mancabelli et al., 2017). The abundance of this genus was positively correlated with colonic TLR4 and TLR9 expression, suggesting a pathogenic loop in which the proliferation of *Erysipelatoclostridium* may increase microbial products that cross the damaged barrier and activate TLR4 and TLR9 on immune cells (Lin et al., 2025). Activation of these receptors is known to trigger downstream signaling pathways, including MyD88/NF-κB, leading to the release of pro-inflammatory cytokines and promoting neutrophil infiltration, thereby exacerbating inflammation. In line with this mechanism, the pro-inflammatory mediators TNF-α, IL-6, CXCL-1, and MPO were elevated and positively correlated with *Erysipelatoclostridium* abundance in our model (Chow et al., 1999; Poltorak et al., 1998; Chen et al., 2024; Berkowitz et al., 2013). MR administration appeared to interrupt this cycle by concurrently reducing *Erysipelatoclostridium* abundance, TLR4/TLR9 expression, and the levels of these pro-inflammatory cytokines. Concomitantly, MR treatment promoted the proliferation of beneficial bacteria. The relative abundances of *Bacteroides* and *Alloprevotella*, known producers of short-chain fatty acids (SCFAs), were increased and showed negative correlations with TLRs and pro-inflammatory factors. SCFAs play a crucial role in enhancing intestinal barrier function by promoting the expression of tight junction proteins, particularly OCC and ZO-1, which is consistent with our immunohistochemistry findings (Macfarlane and Macfarlane, 2012; Jung et al., 2015; Peng et al., 2009).

### 4.2 MR restores intestinal barrier integrity by upregulating tight junction proteins

The intestinal barrier is crucial for maintaining intestinal homeostasis and preventing the translocation of detrimental luminal substances. Its structural integrity is primarily maintained by the tight junction (TJ) complex, which comprises key proteins such as Claudins, OCC, and ZO. The expression levels of these TJ proteins are a critical determinant of intestinal permeability (Cobo et al., 2017; Schmitz et al., 1999). Consistent with this principle, our study showed that DSS-induced colitis was associated with a marked reduction in OCC and ZO-1 expression, accompanied by increased intestinal permeability, as indicated by elevated FITC-DEX levels. This compromised barrier function permits the translocation of luminal pathogens and toxins, which in turn elicit immune activation and the release of pro-inflammatory cytokines such as TNF-α. As reported in the literature, TNF-α can activate myosin light chain kinase (MLCK), promoting cytoskeletal contraction, disrupting TJ integrity, and thereby further increasing intestinal permeability in a vicious cycle (Kishida et al., 2015; Gomaa, 2020; Ghouri et al., 2020). Our correlation analysis supports this pathological framework, showing that the abundance of *Erysipelatoclostridium* was negatively correlated with OCC and ZO-1 expression, suggesting that this pathogenic genus may impair barrier function by releasing toxins and inducing pro-inflammatory responses. In contrast, the beneficial genera *Bacteroides* and *Alloprevotella* were positively correlated with OCC and ZO-1 levels, likely reflecting their reported role in promoting TJ protein expression through metabolic products such as SCFAs (Huo et al., 2022). Notably, MR treatment effectively counteracted these deficits, leading to increased OCC and ZO-1 expression and a corresponding decrease in FITC-DEX permeability. Collectively, these findings suggest a dual mechanism underlying the therapeutic effects of MR: it helps restore a healthy intestinal environment by suppressing pathogenic bacteria and associated inflammation, while simultaneously promoting the upregulation of TJ proteins, thereby strengthening barrier integrity, reducing permeability, and ultimately alleviating colitis.

### 4.3 Certain compounds in MR can regulate the gut microbiota

The intrinsic phytochemical profile of MR is also likely to contribute to its gut-protective effects. Flavonoids such as quercetin, kaempferol, and chrysin have been shown to strengthen intestinal barrier function by upregulating tight junction proteins (ZO-1, OCC) while concurrently suppressing mucosal inflammation by inhibiting TLR4/NF-κB signaling (Qu et al., 2021; Xiong et al., 2025). These compounds also reshape the gut microbiota in favor of commensal taxa (e.g., elevating SCFA-producing *Ruminococcaceae* and *Prevotellaceae* while reducing *Proteobacteria*), thereby enhancing short-chain fatty acid production and immune homeostasis (Yao et al., 2025). Tannins and phenolic acids likely complement these effects; for example, persimmon-derived condensed tannins have been reported to ameliorate colitis by downregulating macrophage pro-inflammatory gene expression and suppressing the expansion of pathogenic *Enterobacteriaceae* in DSS-treated mice (Kitabatake et al., 2021). In addition, triterpenoids present in MR (e.g., ursolic acid) may further contribute to epithelial protection and resolution of inflammation, as they can activate antioxidant defenses and inhibit NF-κB-driven cytokine release in colitic tissues (Liu et al., 2016). Taken together, these MR-derived constituents appear to reinforce the gut barrier, modulate microbial balance, and attenuate inflammatory responses, thereby jointly contributing to the restoration of intestinal homeostasis in DSS-induced colitis.

### 4.4 MR reverses metabolic dysregulation by modulating key fecal metabolites

The gut microbiota modulates intestinal homeostasis in part through the production of diverse metabolites. Our metabolomics analysis revealed that MR intervention markedly increased the levels of Sanjoinine G2 and several acylcarnitines, including Heptadecanoyl carnitine, 15-Methylheptadecanoylcarnitine, and 5-Methylpentadecanoylcarnitine. These metabolites may contribute to alleviating IBD through various mechanisms. Sanjoinine G2, a bioactive alkaloid with sedative properties, may indirectly improve IBD-related manifestations by mitigating comorbidities such as anxiety and insomnia (Lund et al., 2024). Acylcarnitines are crucial regulators of mitochondrial *β*-oxidation, a key pathway for ATP generation via oxidative phosphorylation (Giesbertz et al., 2015). Notably, the abundances of beneficial bacteria such as *Bacteroides* and *Alloprevotella* were positively correlated with the levels of these acylcarnitines and Sanjoinine G2. Given that colonocyte function is highly dependent on mitochondrial energy and that these genera can supply additional energy substrates through the production of SCFAs, these correlations suggest that MR may help restore intestinal barrier integrity by enriching short-chain fatty acid–producing bacteria and upregulating metabolites related to mitochondrial *β*-oxidation. In contrast, MR treatment led to a significant reduction in the levels of Bethanidine and 2-Oxopopulifolic acid. Bethanidine inhibits norepinephrine (NE) release from sympathetic nerve endings, thereby reducing sympathetic tone. This is particularly relevant because dysregulation of sympathetic nerve activity, with consequent impairment of intestinal microcirculation, has been implicated in the pathogenesis of IBD-related intestinal ischemia (Grunz et al., 2023). Although the biological role of 2-Oxopopulifolic acid remains unclear, its elevation in the DSS model may reflect increased release of metabolites associated with microbial dysbiosis or epithelial damage, potentially implicating it in IBD pathogenesis. Furthermore, both Bethanidine and 2-Oxopopulifolic acid were positively correlated with the pathobiont *Erysipelatoclostridium*. Thus, MR treatment may help protect the intestinal barrier in part by reducing Bethanidine levels, thereby modulating sympathetic nerve function and improving intestinal perfusion, whereas the concurrent decrease in 2-Oxopopulifolic acid suggests a potential biomarker whose biological significance warrants further investigation. Pathway analysis revealed upregulation of the Neurotrophin signaling pathway in the DSS group. Activation of this pathway via the p75NTR signaling molecule enhances the expression of pro-inflammatory factors such as IL-6 through the TRAF6-IKKα/β axis, a mechanism implicated in chronic inflammatory diseases (Bandoła et al., 2017). This suggests that heightened activity of this pathway in the DSS group contributed to increased release of pro-inflammatory mediators and exacerbation of the inflammatory response. Conversely, MR treatment markedly downregulated pathways, including those involved in Pyrimidine metabolism. This is consistent with previous reports showing that dysregulated Pyrimidine metabolism aggravates intestinal epithelial cell apoptosis and compromises barrier function in other models of intestinal inflammation (Fu et al., 2022). In our study, epithelial apoptosis was not directly assessed; therefore, the observed modulation of Pyrimidine metabolism by MR should be interpreted as a potential mechanism contributing to intestinal barrier protection, which requires further experimental validation.

### 4.5 Limitations and future perspectives

This study provides experimental evidence that the traditional Chinese medicine MR alleviates DSS-induced colitis through a multi-pronged mechanism involving regulation of the gut microbiota, correction of metabolic imbalances, and restoration of intestinal barrier integrity. Despite these promising findings, several limitations of the present study merit consideration. First, our results are primarily correlational and do not establish causality between the observed microbial and metabolic shifts and the therapeutic outcome. For example, elucidating the precise roles of key metabolites such as Sanjoinine G2, Bethanidine, and 2-Oxopopulifolic acid will require future mechanistic studies, including those involving exogenous supplementation or targeted blockade. Second, regarding innate immune signaling, we focused on TLR4 and TLR9 as representative pattern-recognition receptors and did not examine other TLRs, such as TLR2, which recognizes Gram-positive bacterial components. Given the predominance of *Firmicutes* and the changes observed in specific beneficial and pathobiont genera in our microbiota data, the lack of TLR2 (and other TLRs) measurements represents an additional limitation that should be addressed in future work. Third, although our pathway analysis implicates the Neurotrophin signaling and Pyrimidine metabolism pathways in IBD pathogenesis, the specific molecular targets and regulatory networks remain to be elucidated. Future multi-omics approaches integrating transcriptomics and proteomics will be necessary to dissect these complex mechanisms. Fourth, the long-term stability of MR-induced microbial alterations and the treatment’s efficacy across different stages of IBD progression (e.g., induction versus remission) warrant further investigation. Finally, given that this study was conducted using an animal model, the applicability of our findings to human clinical practice requires further investigation.

## 5 Conclusion

MR attenuates the progression of inflammatory bowel disease (IBD) by reshaping the intestinal environment. It rebalances the intestinal microbiota, restores intestinal barrier integrity by upregulating tight junction proteins, suppresses TLR4/TLR9 expression, and corrects metabolic disturbances. In doing so, MR helps to interrupt the vicious cycle linking dysbiosis, barrier dysfunction, and aberrant immunity that perpetuates chronic intestinal inflammation. This multi-target mode of action may offer advantages over single-target biological inhibitors. As a traditional therapeutic agent with a favorable safety profile, MR represents a promising candidate for the development of safe and effective long-term treatment strategies for IBD.

## Data Availability

The data presented in this study are publicly available. The data can be found here: https://www.ncbi.nlm.nih.gov, accession PRJNA1373134.
